# An unexpected overlap syndrome of mitral valve prolapse with COVID-19 related myocarditis: case report from two patients

**DOI:** 10.1097/MS9.0000000000000522

**Published:** 2023-04-03

**Authors:** Mochamad Y. Alsagaff, Khubay A. Shonafi, Saskia D. Handari, Yan E. Sembiring, Terrence T. E. Lusida, Ricardo A. Nugraha

**Affiliations:** a Department of Cardiology and Vascular Medicine; b Department of Thoracic Cardiac and Vascular Surgery, Faculty of Medicine, Universitas Airlangga – Dr. Soetomo General Hospital; c Department of Cardiology, Faculty of Medicine, Ciputra University, Surabaya, Indonesia

**Keywords:** COVID-19, mitral valve prolapse, myocarditis

## Abstract

The authors reported two patients with a history of asymptomatic mild mitral valve prolapse, a male in his late 40s (Case 1, vaccinated) and a female in her late 20s (Case 2, unvaccinated), who developed worsening (severe) mitral prolapse and New York Heart Association symptoms class III–IV after exposure to coronavirus disease 2019 with evidence of myocarditis on MRI. Both patients received similar 6-month of heart failure therapy; however, the outcomes did not affect the severity of their symptoms or mitral regurgitation. Subsequently, both patients underwent mitral valve surgery.

## Background

HIGHLIGHTS

- Myocarditis or inflammation in coronavirus disease 2019 (COVID-19) can worsen mitral valve prolapse.
- COVID-19 infection and complications are still possible following COVID-19 exposure without a positive swab test (nonbreakthrough case).
- Inflammatory markers and MRI imaging can be used to confirm the resolution of the inflammatory process in COVID-19 related myocarditis and mitral valve regurgitation, as well as to determine the possible timing of mitral valve surgery.

Myocarditis in the setting of coronavirus disease 2019 (COVID-19) infection is a clinical issue that has received much attention. It is plausible that COVID-19 infects myocardium, especially in patient with a previous history of hypertension or heart failure, as angiotensin converting enzyme 2 is upregulated, although the presence of viral receptors does not always predict tropism^1^. The incidence of COVID-19 related myocarditis is ~7% and it is contributed to the COVID-19 related mortality^2^.

While the acute inflammation and injury to the myocardium are the current focus receiving attention, the effects of exacerbation of previous existing valvular heart diseases are completely unknown. Mitral valve prolapse might be secondary, caused by dilatation of the mitral annulus usually seen in the myocarditis or directly due to the postendomyocarditis lesion of the mitral apparatus^3^. A previous case report described the histopathological finding of the mitral valve prolapse in the setting of COVID-19 infection, which revealed myxomatous degeneration with an inflammatory infiltrate composed of T lymphocytes and histiocytes. Immunohistochemistry identified these T cells as the CD4 helper subtype without any CD8+ T cells^4^.

Interestingly, exposure to COVID-19 in case A with a negative swab test and in case B with a positive swab test resulted in an identical clinical and imaging course. The message is that, while Case A is not a breakthrough case, the inflammation in myocarditis continues as in Case B. Hereby, we present two cases of acute myocarditis during the start of the COVID-19 pandemic and discuss how valvular severity evolves from mild to severe following myocardial edema. This case highlights the risk of COVID-19 infection leading to acute myocarditis and severe mitral prolapse. To our knowledge, this is the first documented case of mitral valve prolapse in the setting of COVID-19 infection. The work has been reported in line with the CARE 2020 Criteria^5^.

## Case A

A 49-year-old man was taken to the hospital with shortness of breath. He had a close contact history with three COVID-19 patients who were hospitalized at the same time. He had a 2-year history of asymptomatic mild mitral valve prolapse and was on hypertension medication.

On physical examination, he had normal blood pressure (110/60 mmHg), normal temperature (37 °C), tachycardia (111 beats/min), mild dyspnea with two-thirds bilateral basilar rales (30 breaths/min), elevated jugular venous pressure, and an apical systolic murmur. Because the patient was desaturated and could not lie on his back, supplemental oxygen at a rate of 15 l/min was administered through a nonrebreathing mask (oxygen saturation of 98%). He already had five nasopharyngeal reverse transcription polymerase chain reaction (RT-PCR) swabs and a rectal swab that were all negative for COVID-19 infection. Two months ago, the patient was vaccinated against COVID-19 twice with Sinovac (Coronavac).

**Figure 1:**
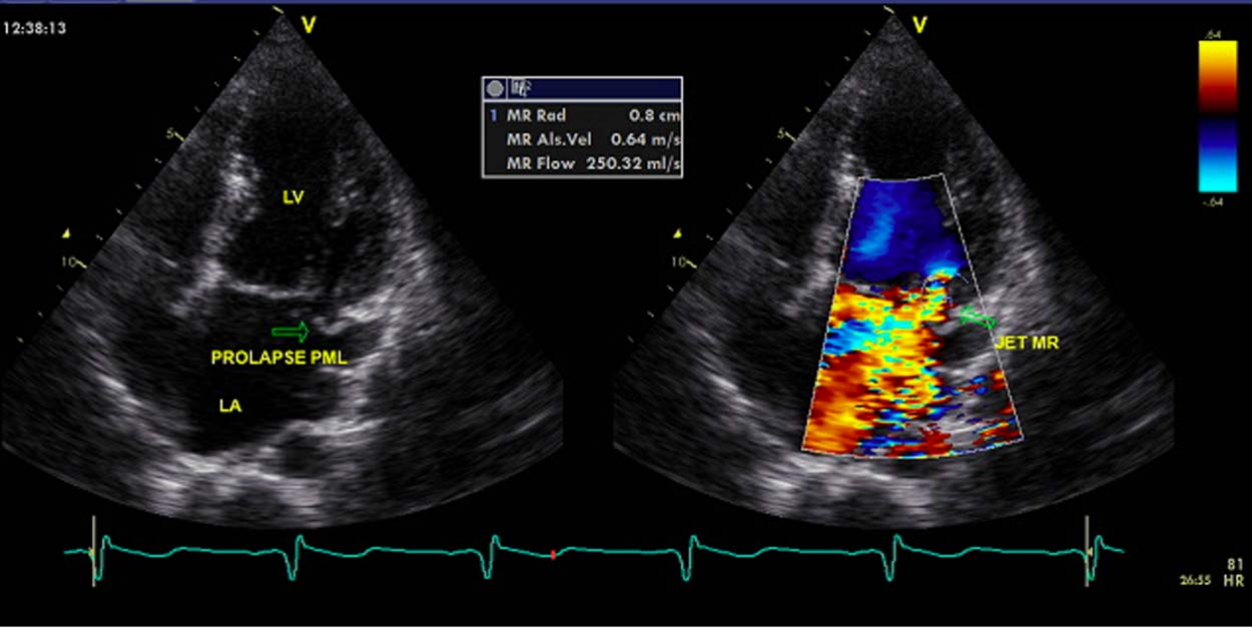
Patient 1 echocardiography. Echocardiography showed left ventricular dilatation, preserved ejection fraction, and severe mitral regurgitation with PML prolapse.

**Figure 2:**
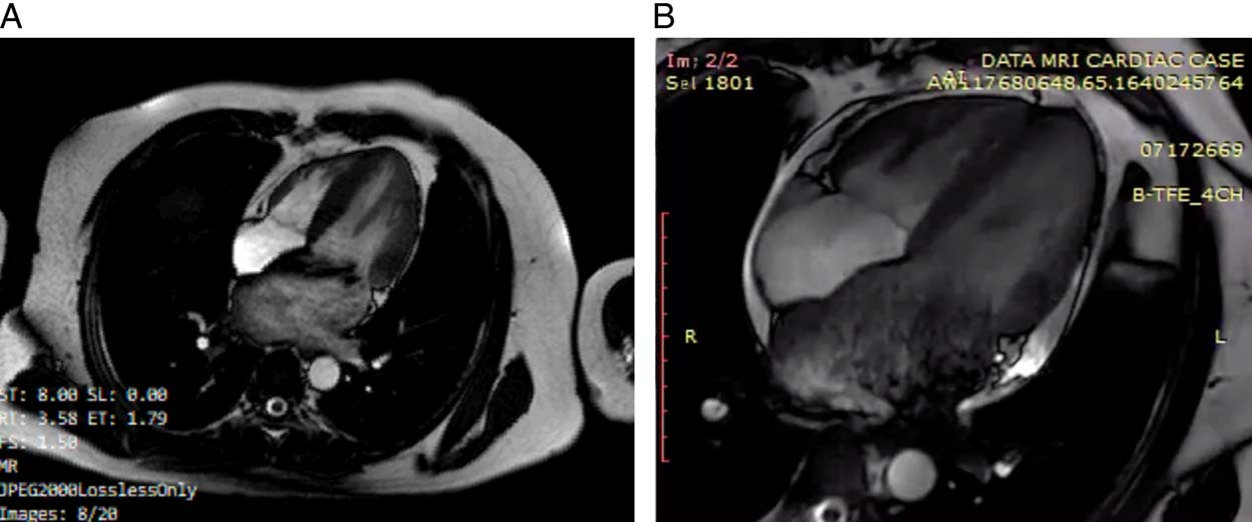
CMR imaging on initial and follow-up presentation for Patient 1. (A) First CMR showed acute myocarditis-related myocardial edema with no evidence of myocardial fibrosis in the inferolateral and apical septal walls. (B) A CMR evaluation revealed diminished cardiac edema without the presence of fibrotic signs.

Routine sputum, urine, and blood cultures were not favorable for any growth of bacterial infections. The chest computed tomography revealed pleural thickening and central ground-glass opacity as well as bilateral lobe consolidation, indicating acute lung edema. Ultrasound echocardiography showed left ventricular dilatation (6.4 cm), preserved ejection fraction (76%), and severe mitral regurgitation with posterior mitral leaflet prolapse (Fig. 1). Cardiac magnetic resonance (CMR) imaging revealed acute myocarditis-related myocardial edema with no evidence of myocardial fibrosis in the inferolateral and apical septal walls (Fig. 2).

Acute heart failure, acute myocarditis, and severe mitral regurgitation (MR) was diagnosed. Due to progressive respiratory distress, he was admitted to the cardiac intensive care unit and intubated for mechanical ventilatory support. The patient was treated with furosemide (5 mg/h), enoxaparin (0.6 mg Once daily(OD)), valsartan (40 mg OD), and spironolactone (25 mg OD). On the seventh day, chest radiography still showed multiple patchy opacities, which had diminished 4 days later. After 13 days, the patient’s condition had improved, and he was extubated. The patient recovered and was discharged on the day-18.

**Figure 3:**
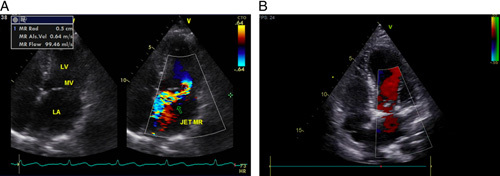
Patient 1 6 months evaluation and post-MV repair echocardiography. (A) Echocardiography evaluation revealed severe MR; (B) echocardiography post-MV repair.

Heart failure therapy was started during the hospitalization and continued in an outpatient setting for 6 months. Unfortunately, despite optimal heart failure therapy, the patient felt that his symptoms worsened with activity (clinical New York Heart Association III–IV). A CMR evaluation was performed on the patient, which revealed diminished cardiac edema without fibrotic signs (Fig. 2). However, the echocardiography evaluation still revealed severe MR (Fig. 3). Therefore, mitral valve (MV) repair was deemed necessary (Fig. 3). Surgical findings showed no evidence of myocarditis in the myocardium or endocarditis in the mitral valve. Seven days after successful surgery, he was discharged without difficulties.

## Case B

A 30-year-old female was taken to a general practitioner with a 7-day history of malaise, cough, fever, and dyspnea. She had a history of asymptomatic mild mitral valve prolapse for 2 years and was not on any heart failure medication. She had been taken to the hospital by her mother around 10 days before because she was infected with COVID-19. The doctors eventually advised the patient to get an RT-PCR swab test, and the findings were positive. Subsequently, the patient decided to self-quarantine at home. The patient did not feel acute shortness of breath during her isolation. Following 14 days of self-quarantine, negative findings of patient swabs were obtained.

**Figure 4:**
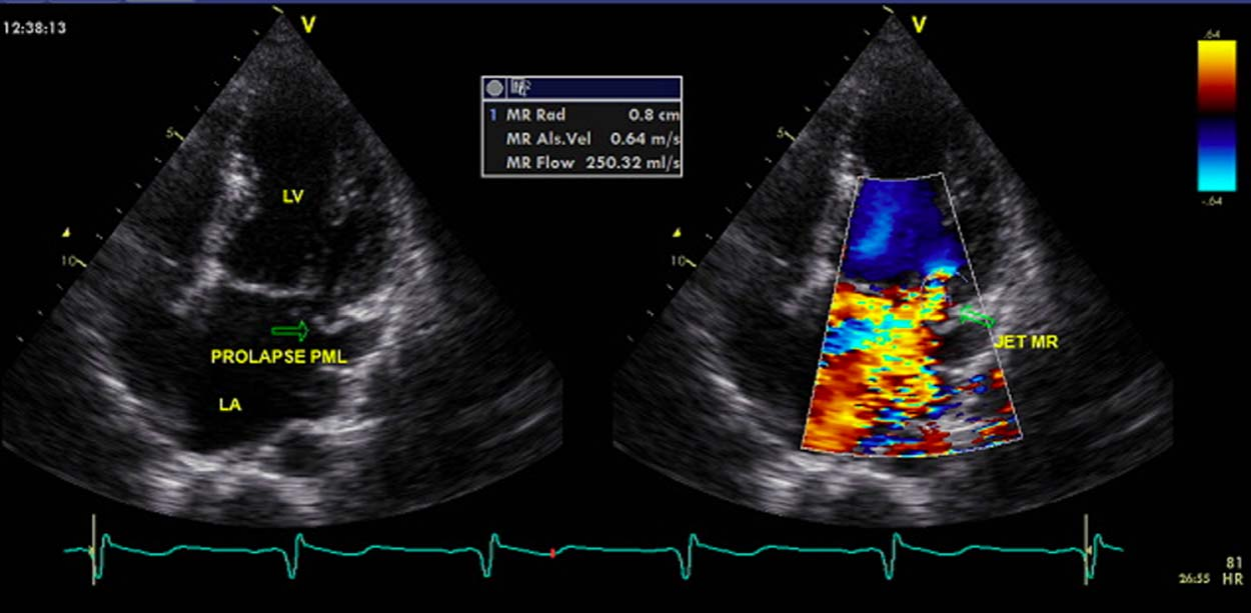
Patient 2 echocardiography. Echocardiography showed left ventricle, preserved ejection fraction, and severe MR with PML prolapse.

**Figure 5:**
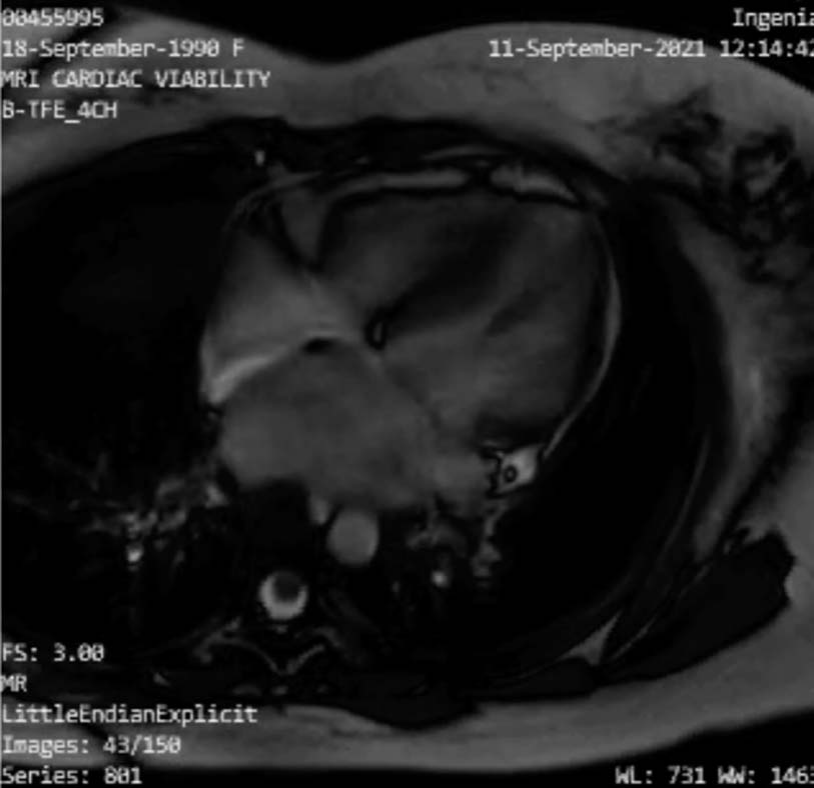
Patient 2 CMR imaging. Late gadolinium improvement exhibited no overdue hyperenhancement in all myocardium.

**Figure 6:**
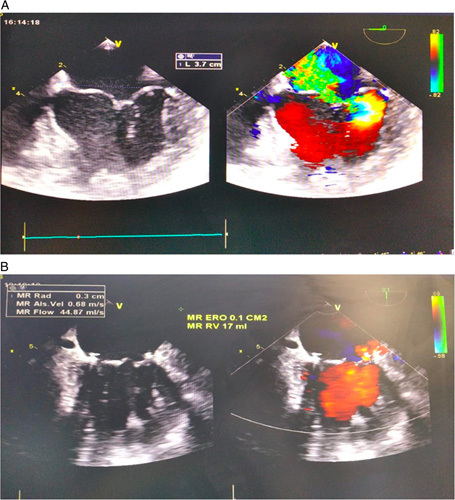
Patient 2 6 months evaluation and post-MV repair echocardiography.

After recuperating from COVID-19, she found herself quickly exhausted and out of breath when engaging in activities. At the cardiologist’s office, an echocardiogram revealed left ventricle dilatation (5.6 cm), preserved ejection fraction (EF 76%), and severe MR with posterior mitral leaflet prolapse (Fig. 4). She was advised to have a CMR examination, which showed myocardial edema related to acute myocarditis without any sign of myocardial fibrosis in the anterior walls basal-mid-apical. Early gadolinium enhancement showed no intracardiac thrombus. Late gadolinium improvement exhibited no overdue hyperenhancement in all myocardium (Fig. 5). She was given heart failure medications (bisoprolol 2.5 mg OD and ramipril 2.5) and was scheduled for MV repaired surgery. After 6 months of waiting for the inflammation to recover (HS CRP decreased from 12.8 mg/l to 5.17 mg/l), the patient underwent mitral valve repair. Surgical findings revealed no evidence of myocarditis in the myocardium or endocarditis in the mitral valve. Seven days after her successful operation, she was discharged without complications (Fig. 6).

## Discussion

We present two unique cases of acute myocarditis with severe mitral valve regurgitation (prolapse) during the COVID-19 pandemic. These patients had a history of asymptomatic mild mitral valve prolapse without functional limitations in the past 2 years. Because of COVID-19 infection, both patients were reported to have severe mitral valve prolapse and evidence of myocardial edema in CMR, consistent with myocarditis. The first case exhibited clinical respiratory failure and pulmonary edema, but no confirmed COVID-19 RT-PCR swab test result. Meanwhile, the second case had a positive COVID-19 RT-PCR swab test but did not have a severe clinical condition. Despite the absence of recurrent infection or myocarditis over the 6-month follow-up period, both patients experienced increased shortness of breath with no signs of reversibility from severe to a lesser degree of mitral regurgitation. Eventually, both patients required surgery to correct their prolapsed mitral valves.

Myocardial edema is a significant aspect of the inflammatory response in acute myocarditis. Suspected myocarditis is one of the most common indications for CMR, with diagnostic findings including myocardial edema, hyperemia, and irreversible injury (necrosis or scar). When clinically suspected, a single positive criterion can aid in diagnosing myocardial inflammation^6^,^7^.

According to a Chinese cohort study, COVID-19 infection can cause 20% of cardiac injuries. Patients with COVID-19 related cardiac injury had a higher percentage of noninvasive and invasive mechanical ventilation (46.3 vs. 3.9% and 22 vs. 4.2%, *P*=0.001), and higher mortality than patients without cardiac injury (51.2 vs. 4.5%; *P*=0.001)^8^. The symptoms and signs of COVID-19 related myocarditis are similar to those of other viral myocarditis. As a result, diagnosing COVID-19 related myocarditis solely based on cardiac symptoms may be challenging, and the presence of additional presentations and clinical involvements might aid in making a diagnosis^9^.

Myocarditis in COVID-19 is still possible in the absence of a positive PCR swab test (nonbreakthrough case). We first thought that the second vaccination dosage caused the first patient’s myocarditis. However, given that the infection occurred 3 months after the last vaccination and the clinical pulmonary edema occurred 1 week later, we concluded that the pulmonary edema was caused by COVID-19 rather than a vaccine side effect.

Despite a negative COVID-19 RT-PCR, the first patient’s symptoms, close history of contact with positive COVID-19 patients, positive COVID-19 antigen, and laboratory findings were all highly suggestive of a 2019 coronavirus disease. As a result, the patient’s cardiac presentation might be related to the COVID-19 infection^10^. Due to virus genome mutations, sampling time, infection severity, sampling location, specimen collection, and test material storage procedures, false-negative RT-PCR testing may happen^11^,^12^. Li *et al*. observed a false-negative rate of 20% when using RT-PCR. If the RT-PCR swab test came out negative, chest imaging would be crucial in making a diagnosis^13^. The first patient had high levels of myocardial injury markers such as NT-proBNP, HS Troponin, and IL-6 (Table 1). NT-proBNP and troponin levels were found to be elevated in 194 COVID-19 related myocarditis cases. Elevated IL-6 suggests the possibility of cytokine storms leading to myocardial edema^14^. Other inflammatory markers, such as CRP and procalcitonin, were elevated and later improved.

**Table 1:** Laboratory results of the first patient.

	Result						
Measure	Day 1	Day 3	Day 5	Day 7	Day 11	Day 15	Day 1
White blood cell	9.800	14.900	12.180	15.790	16.020	26.490	11.910
Neutrophil count	76.4	92.1	87.7	84.2	83.9	88	72.9
Lymphocyte count	16.2	5.2	4.8	8.8	5.4	7.2	17.6
Erythrocyte sedimentation rate	34	37	30	50	45		
Blood urea nitrogen	13		29		36	40	19
Creatinine	1		0.9		1.1	0.9	1.2
Sodium	137		138		134	131	134
Potassium	3.6		3.5		4.7	5.2	5.2
Chloride	103		99		96	99	98
C-reactive protein	13.4			107	60	37.1	25.2
D-Dimer	2.57			1.18	1.51		
High-sensitivity Troponin		14.3					
NT-Pro BNP					272		
Procalcitonin	0.04		0.02				
IL-6				62.68			
COVID-19 test	RT-PCR –	Antigen+	RT-PCR –		Rectal PCR swab –		

Two potential pathophysiology of COVID-19 related cardiac injuries include ACE2 receptor-mediated myocardial injury and a cytokine storm caused by an imbalanced immunological response^7^,^15^. ACE2 receptors have been discovered in myocardial tissue and cardiac valves. Research has shown that the downregulation of the ACE2 receptor axis may promote fibrosis and inflammation in heart valves, leading to insufficiency^16^,^17^. We are confident that the posterior leaflet prolapses were due to the damage caused by massive cytokine storms and immune cells. Autopsies performed on COVID-19-positive patients revealed the same case as well as inflammation on the prior mitral valve chordae apparatus^18^. Based on the evidence from past autopsy reports and case studies on COVID-19-induced myocarditis, we concluded that our patients’ acute mitral valve regurgitation was caused by COVID-19-induced myocardial injury^4^. The mitral valvular lesion is characterized by the leaflets and chordal thickening and degeneration. As regurgitation progresses, the mitral annulus gradually dilates. An increase in prolapse may further stretch and damage the chordae tendineae^19^.

Until now, we have yet to find myocarditis caused by COVID-19 in patients with mitral valve prolapse. We had hoped that once the inflammation subsided, the patient would revert to a mild MR as before the exposure (functional MR). It turned out that the severe MR persisted, and both patients felt confined in their inactivity and inability to work. When the patients’ inflammation subsided (confirmed by laboratory markers and imaging), it was discovered that the severe MR remained. As a result, in symptomatic cases of severe MR, surgical MV repair is the definitive therapy^20^,^21^. Following surgery, both patients have resumed their pre-COVID activities (New York Heart Association symptoms class I) and are on beta-blocker and anticoagulant medication.

## Conclusion

In summary, we suspected that COVID-19 related myocarditis and subsequent severe mitral regurgitation are caused by various pathogenic mechanisms, such as a massive cytokine storm, immune cell-mediated inflammation, and ACE2 receptor downregulation. These pathological processes cause valvular insufficiency by inducing inflammation and fibrosis in the heart valves. Based on prior autopsy reports and COVID-19-induced myocarditis studies, we conclude that our patient’s severe mitral valve prolapse was caused by COVID-19-induced myocardial injury. Acute myocarditis resolves in around 50% of cases within the first 2–4 weeks, with the remaining 12–25% deteriorating or progressing to the final stage of dilated cardiomyopathy. On the other hand, severe mitral regurgitation will not be reversible and will require surgical repair.

## Ethical approval

No ethical approval is needed for this case series.

## Consent for publication

Written informed consent was obtained from the patients for publication of this case report.

## Sources of funding

This article is no funded by any organization or institution, it is granted as case series publication from the hospital.

## Conflicts of interest disclosure

The authors declare no conflict of interest.

## Data availability statement

The datasets used and/or analyzed during the current study are available from the corresponding author on reasonable request.

## Provenance and peer review

Not commissioned, externally peer reviewed.
